# A *Listeria monocytogenes* clone in human breast milk associated with severe acute malnutrition in West Africa: A multicentric case-controlled study

**DOI:** 10.1371/journal.pntd.0009555

**Published:** 2021-06-29

**Authors:** Marièma Sarr, Maryam Tidjani Alou, Jeremy Delerce, Saber Khelaifia, Nafissatou Diagne, Aldiouma Diallo, Hubert Bassene, Ludivine Bréchard, Vincent Bossi, Babacar Mbaye, Jean-Christophe Lagier, Anthony Levasseur, Cheikh Sokhna, Matthieu Million, Didier Raoult

**Affiliations:** 1 IHU-Méditerranée Infection, Marseille, France; 2 Aix Marseille Univ, IRD, AP-HM, MEPHI, Marseille, France; 3 Campus Commun UCAD-IRD of Hann, Dakar, Senegal; 4 Aix Marseille Univ, IRD, AP-HM, VITROME, Marseille, France; NIH-National Institute for Research in Tuberculosis-ICER, INDIA

## Abstract

**Background:**

Severe acute malnutrition (SAM) is a major public health problem affecting children under the age of five in many low- and middle-income countries, and its resolution would contribute towards achieving the several sustainable development goals. The etiology of SAM is pluri-factorial, including delayed maturation of the gut microbiota, suboptimal feeding practices and dysfunctional breastfeeding. The recent serendipitous detection of *Listeria monocytogenes* in the breast milk of Malian women, in contrast to French women, suggests a possible association with SAM.

**Methodology/ Principal findings:**

To investigate the possible association of *L*. *monocytogenes* carriage in breast milk and SAM, a case-control study was performed in Senegal, with subjects recruited from two areas. Using 16S amplicon sequencing, a culture independent method, 100% (152/152) of the mothers were positive for *L*. *monocytogenes* in their breast milk while qPCR analysis gave lower recovery rates. Interestingly, after enrichment in Fraser broth and seeding on PALCALM agar, all 10 isolated strains were isolated from the milk of 10 mothers who had SAM children which also had a significantly increased relative abundance of *L*. *monocytogenes* (0.34 (SD 0.35) vs 0.05 (SD 0.07) in controls, p<0.0001). The high genomic similarity between these strains and Malian breast milk strains from a previous study supports the hypothesis of endemic clone carriage in West Africa. Moreover, the *in vitro* growth inhibition of *L*. *monocytogenes* using breast milk samples was obtained from only 50% of the milk of mothers who had SAM children, in contrast to control samples which systematically inhibited the growth of *L*. *monocytogenes* with a higher inhibition diameter (15.7 mm (SD 2.3) in controls versus 3.5 mm (SD 4.6) in SAM, p = 0.0001). *Lactobacillus* and *Streptococcus* isolated from the breast milk of controls inhibit *L*. *monocytogenes* in a species-dependent manner.

**Conclusions/Significance:**

Our study reveals a previously unsuspected carriage of *L*. *monocytogenes* in the breast milk of West African women, which is associated with SAM. The inhibitory effect of human selected lactic acid bacterial species against *L*. *monocytogenes* might provide new therapeutic and inexpensive options to prevent and treat this neglected public health issue.

## Author summary

Severe acute malnutrition is a global public health issue which greatly impacts childhood mortality rates. Although still not fully understood, the multi-factorial pathology of severe acute malnutrition has been associated, among other factors, with sub-optimal feeding practices (including dysfunctional breastfeeding) and an altered gut microbiota. The serendipitous detection of *Listeria monocytogenes* in the breast milk of Malian women has raised the possibility of its involvement in the pathogenesis of severe acute malnutrition. To investigate this possibility, the presence of *L*. *monocytogenes* was assessed in a cohort of lactating Senegalese women, both mothers of healthy children as well as those of severely malnourished children using culture-dependent and independent methods. Our study confirms the previously unsuspected presence of *L*. *monocytogenes* in the breast milk of Senegalese women, which is increased in the milk of mothers of severely malnourished children. Moreover, breast milk samples from the mothers of healthy children more frequently induced a potent inhibition of *L*. *monocytogenes* than those from the mothers of severely malnourished children. An inhibition was also achieved using potential probiotics, *Lactobacillus* and *Streptococcus* species, isolated from breast milk. Our study reveals the previously unsuspected carriage of *L*. *monocytogenes* in the breast milk of West African women, which is associated with severe acute malnutrition. The inhibitory effect of human selected lactic acid bacterial species against *L*. *monocytogenes* might provide new therapeutic and inexpensive options to prevent and treat this neglected public health issue.

## Introduction

Severe acute malnutrition (SAM) is a major public health issue with major consequences for childhood mortality rates [1]. SAM affects about 16 million children under the age of five around the world at any given time with a prevalence of 2.4% [2], leading to a four to eight fold increased death rate compared to that of well-nourished children [3]. In West Africa specifically, nearly eight million children suffer from SAM [4]. This severe form of childhood malnutrition is associated with inadequate food intake, malabsorption and infection which alters the metabolism and weakens the immune system [5]. There are two forms of SAM, distinguished by the presence of a nutritional bilateral oedema: kwashiorkor (oedematous SAM) and marasmus characterized by extreme wasting (non-oedematous SAM) [6].

The instrumental role of the alteration of the intestinal microbiota has recently been highlighted in this context [7]. The etiology of SAM and particularly kwashiorkor cannot be solely explained by nutritional deficiency. A pioneering study in twins discordant for kwashiorkor has revealed delayed maturation of the intestinal microbiota, and demonstrated that the “weight loss” phenotype could be transmitted to gnotobiotic mice through faecal microbiota transplantation combined with the local diet [8]. Since then, several studies have shown an alteration of the intestinal microbiota in severely malnourished children characterized by a decrease in overall diversity [9,10], increased colonization of the digestive microbiota by pathogenic species (*Proteobacteria*, *Fusobacteria*, *Enterococcus faecalis*, *Escherichia coli*, *Staphylococcus aureus*), and a depletion of oxygen-intolerant species, particularly a loss of methanogenic archaea [9,10]. In addition, this depletion in oxygen-intolerant species is associated with an oxidation of the digestive environment [9], as previously described [11]. More recently, our team has identified potential probiotics to restore missing gut microbes [10] and has highlighted a difference between marasmus and kwashiorkor with a specific increase in *Proteobacteria*, *Fusobacteria* and pathogens in kwashiorkor [12].

The earliest studies describing SAM and, more specifically, kwashiorkor have mentioned dysfunctional breastfeeding and weaning as etiological factors in the pathogenesis of SAM [13]. Breast milk contains a plethora of distinct bioactive molecules which protect against infection and inflammation and contribute towards immune maturation [14]. Moreover, recent insights into the composition of the breast milk and tissue microbiota have revealed a diverse ecosystem [15]. Recent studies have highlighted the existence of a vertical transfer of microbiota through breast milk [16]. However, the few studies regarding vertical transfer have seldom described pathogen transmission. *Listeria monocytogenes* was first reported in human breast milk in 1988 [17]. Since then, only one study by our group showed a prevalence of *L*. *monocytogenes* in breast milk. A serendipitous finding, while exploring the microbial diversity of breast milk, highlighted a prevalence of *L*. *monocytogenes* in the breast milk of Malian mothers from an area known for endemic malnutrition [18], while healthy French lactating women did not present any *L*. *monocytogenes* in their breast milk. This well-known pathogen is the agent of listeriosis, a food-borne disease that includes maternal-neonatal infections possibly leading to foetal loss [19]. Interestingly, maternal listeriosis was three times more prevalent in pregnant women born in Africa than in the French general population [19].

The present study is not only aimed at confirming the carriage of *L*. *monocytogenes* in the breast milk of lactating women from West Africa but also how this observation might be associated with SAM, with a dysbiosis of the breast milk microbiota. We therefore conducted a two-centre case-control study in Senegal to investigate the prevalence of *L*. *monocytogenes* in the breast milk of these women and its possible association with SAM, using culture-dependent and independent analyses.

## Material and methods

### Ethics statement

The study obtained approval from the Senegalese National Ethics Committee for Health Research (CNERS) (Protocol SEN16/45), 7 March 2017. Informed and signed consent was obtained from all participants.

### Design and sampling

This case-control study was carried out with groups with similar age and sex distribution according to the STROBE statement [20] check list (S1 STROBE Checklist). Breastfeeding mothers of severely malnourished and healthy children were recruited in Senegal over two periods, between August and December 2017, and between September and December 2018 in the Notre Dame Esperance clinic in Thiaroye, located in a suburban area of Dakar (cases n = 90 and controls n = 22), in the medical centres in Toucar, Ngayokheme and Diohine, and in the public and “private” health centres located in the Fatick region of the Niakhar municipality (cases n = 30 and controls n = 10), 150 km from the Dakar region. Milk samples (100μL to 500μL) were collected in sterile cryotubes after disinfecting the nipple with a saline solution (NaCl 0.85%) and 70% ethanol. 50 to 100 μL of the C-Top liquid conservation medium (Culture Top, Marseille, France), consisting mostly of three antioxidants, namely ascorbic acid, glutathione and uric acid, was added to all samples and stored at 4°C for two to three hours prior to storage at -80°C in the VITROME laboratory (Dakar, Senegal). Samples were then shipped on dry ice to the University Hospital Méditerranée Infection of Marseille for microbiological and molecular analyses.

### Cases and control inclusion

SAM was defined according to the WHO criteria for children aged 6 to 59 months, namely anthropometric criteria (a weight-for-height z-score <-3SD and/or a brachial perimeter less than 115 mm) and/or the presence of bilateral oedema [21]. Controls were mothers of children with no anthropometric deficits according to the criteria defined by WHO [21] and confirmed by the WHO anthro software [22] (version 3.2.2, 2011, http://www.who.int/childgrowth/software/en/). Controls were similar in age to the SAM cases and lived in the same areas. Lactating mothers of the included children suffering from SAM were asked for a milk sample at the time of initial care. Breastfeeding mothers of the control children were asked for a milk sample at inclusion.

Exclusion criteria included delivery by caesarean section, refusal to participate by the parents or the legal guardian, children who were not breastfed, the absence of sample collection, and requests for withdrawal. Mothers of children presenting with respiratory symptoms, malaria, varicella or sickle cell disease were excluded from the study. Mothers of children with diarrhea or fever were excluded from the control group but not from the SAM group, as these symptoms form part of the clinical features of SAM. All the women (mothers of SAM and healthy children) included in the cohort were asymptomatic (no fever, diarrhoea or other symptoms of infection). Anthropometric parameters, namely age, sex, weight, height, and mid-upper arm circumference (MUAC), were collected for all children. The weight was measured using an electronic infant scale (Greetmed, Zhejiang Province, China) while the height was assessed using a height gauge. As for the MUAC, it was measured using the WHO-recommended tape.

### Detection of *Listeria monocytogenes* using culture-independent methods in the milk of Senegalese mothers

#### DNA extraction protocols

Proteins and glycans may hinder DNA extraction, consistently with the fact that chemical lysis using proteinase K and deglycosylation prior to extraction has been reported to induce substantial differences in metagenomic gut studies [23,24]. To optimize the detection of *L*. *monocytogenes*, three DNA extraction protocols (automated extraction, proteinase K treatment prior to automated extraction, deglycosylation followed by proteinase K treatment prior to automated extraction) were compared.

*Automated extraction*. After a 30-minute sonication of all breast milk samples, DNA extraction was performed using an EZ1 Advanced XL automated device (Qiagen, Courtaboeuf, France) and the QIAGEN-DNA kit Tissue (Omega bio-tek, Norcross, GA, USA) with an elution volume of 50 μL. DNA tubes were stored at -20°C until use.

*Deglycosylation and proteinase K treatments prior to automated extraction*. Samples were extracted by a mechanical treatment performed with acid-washed glass beads (Sigma-Aldrich, Saint-Louis, MO, USA) and 0.5mm glass bead cell disruption media (Scientific Industries, Inc., NewYork, NY, USA) using a FastPrep-24 5G Grinder (MP Biomedicals, Illkirch-Graffenstaden, France) at maximum speed (6.5 m/sec) for 90 seconds. This was followed by a chemical lysis, consisting of either an overnight proteinase K treatment or an overnight deglycosylation step followed by a proteinase K treatment [24] and finally, an automated extraction purification.

#### Quantitative polymerase chain reaction (qPCR)

We performed a quantitative PCR (qPCR) specific for *L*. *monocytogenes* on all breast milk samples after DNA extraction. qPCR was performed using the CFX96 Real-Time system (Bio-Rad, Laboratories, Foster City, CA, USA). Reactions were carried out in a final volume of 20 μL including 10μL of master mix (Roche Diagnostics GmbH, Mannheim, Germany), 0.5 μl of each primer per reaction (Fwd and Rev), 0.5 μL of Uracil-DNA glycosylase (UDG), 0.5 μl probe, 3μL of Ultrapure distilled water, DNAse, RNAse-free (Invitrogen, Carlsbad, California, USA) and 5 μL of DNA. The applied protocol was that of Roche, which consists of two minutes at 50°C, five minutes at 95°C followed by 40 cycles of five seconds at 95°C and 30 seconds at 60°C. The results of the qPCR were considered negative in the absence of an amplification curve.

#### 16S rRNA amplicon sequencing

16S rRNA amplicon sequencing was performed from DNA extracted with and without prior deglycosylation followed by proteinase K using MiSeq technology (Illumina Inc., San Diego, CA, USA) with paired-end strategy, constructed according to the 16S Metagenomic Sequencing Library Preparation (Illumina). DNA was amplified for the 16S “V3-V4” regions by PCR for 45 cycles, using the Kapa HiFi Hotstart ReadyMix 2x (Kapa Biosystems Inc., Wilmington, MA U.S.A), and the surrounding conserved region V3_V4 primers with overhang adapters (FwOvAd_341F TCGTCGGCAGCGTCAGATGTGTATAAGAGACAGCCTACGGGNGGCWGCAG; RevOvAd_785R GTCTCGTGGGCTCGGAGATGTGTATAAGAGACAGGACTACHVGGGTATCTAATCC. After purification on AMPure beads (Beckman Coulter Inc, Fullerton, CA, USA), concentration was measured using high-sensitivity Qubit technology (Beckman Coulter Inc., Fullerton, CA, USA) and dilution to 3.5 ng/μl was performed. At this step, the library without deglycosylation was pooled with equal volumes of the library with deglycosylation, and Illumina sequencing adapters and dual-index barcodes were added to the amplicon. After purification on AMPure beads (Beckman Coulter Inc., Fullerton, CA, USA), the first library was pooled with 95 multiplexed samples and the second library with 41 multiplexed samples. The global concentration was quantified by a Qubit assay with the high sensitivity kit (Life technologies, Carlsbad, CA, USA). Before loading for sequencing on MiSeq (Illumina Inc, San Diego, CA, USA) the pool was diluted at 8pM. Automated cluster generation and paired-end sequencing with dual index reads was performed in a single 39-hour run in a 2x250bp. The paired reads were filtered according to the read qualities. The raw data were configured in fastq files for R1 and R2 reads. Operational taxonomy units (OTUs) and taxonomic assignment were performed as reported previously [25].

### Detection of *L*. *monocytogenes* by culture-dependent methods

Culture was carried out by enriching 10 μl of breast milk samples in Fraser broth (Fisher Scientific, Illkirch, France) which were incubated for 48 hours at 37°C. 50 μL of the culture was then seeded in PALCALM agar (Fisher Scientific, Illkirch, France), incubated for between 24 and 48 hours at 37°C before colony growth. The identification of these colonies was achieved using Matrix Assisted Laser Desorption Ionisation—Time of Flight mass spectrometry (MALDI-TOF MS) on a Microflex LT spectrometer (Bruker Daltonics, Heidelberg, Germany).

### Lactograms and antimicrobial assays

The inhibitory activity of breast milk on *L*. *monocytogenes* CSUR Q0781, isolated from the milk of the mother of a SAM child, was tested on 5% sheep blood-enriched Columbia agar (bioMerieux, Marcy l’Etoile, France) and ordinary nutritive agar, with amoxicillin as a positive control and sterile PBS as a negative control. Three holes were punched in the plates after seeding with *Listeria monocytogenes* at a concentration of 0.5 McFarland, which were subsequently filled with 100μL of breast milk from mothers of children suffering from SAM, that from mothers of healthy children, or PBS. Potentially antagonistic species were also tested using the same protocol with a bacterial suspension of 0.5 McFarland.

### Statistics

Statistical analyses were performed using SPSS software version 20.0 (IBM, Paris, France), SAS 9.4 statistical software (SAS Institute, Cary, NC) and GraphPad Prism 9.0 (GraphPad software, La Jolla, USA). To test for a statistical association between the detection of *L*. *monocytogenes* and severe acute malnutrition using qPCR and/or culture, we used the bilateral Barnard exact test [26]. Quantitative data distribution was assessed using the Kolmogorov-Smirnov and Shapiro-Wilk normality tests. Quantitative variables were compared using the bilateral Student test or Mann-Whitney test depending on the data distribution assessed by the normality test. All tests were two-tailed with a significance threshold set at a value of p≤ 0.05.

## Results

### Population

**Fig 1:**
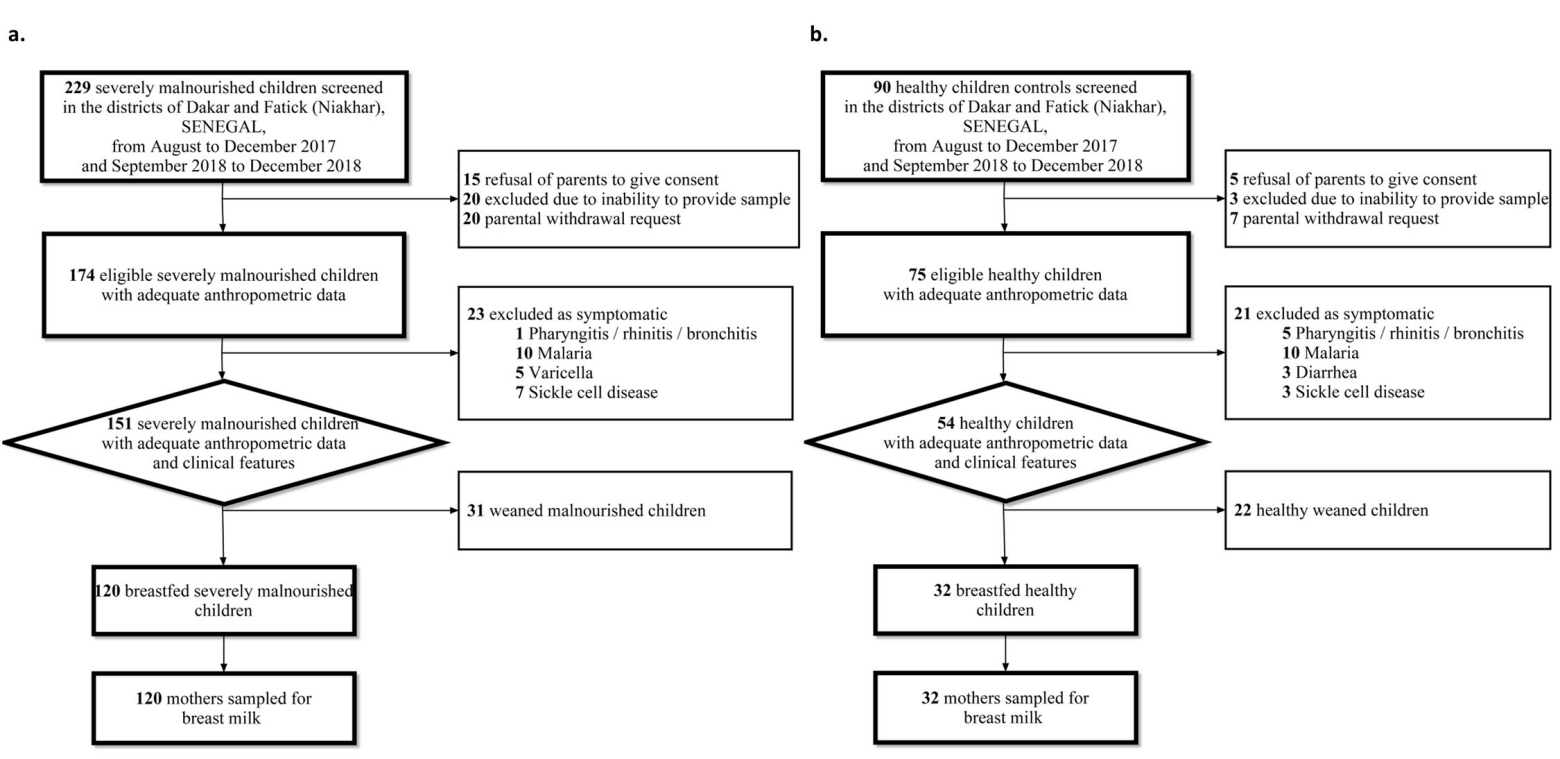
Selection of the study population with (a) case selection based on exclusion criteria including lack of ethical approval, WHO anthropometric and clinical criteria and weaning (b) control selection based on exclusion criteria including lack of ethical approval, weaning and symptoms.

Analysis of the baseline characteristics (Table 1) did not reveal any statistical difference between the ages of the cases and controls, nor that of the mothers. As expected, anthropometric indicators were significantly lower in the SAM cases than in the controls. Besides nutritional indicators, we also observed that fever and diarrhoea were frequent in the SAM cases (respectively 81.6% and 77.5%). We screened 229 severely malnourished children, of whom 174 had anthropometric measurements consistent with SAM or presented with nutritional oedema. 23 children who presented pharyngitis, rhinitis, bronchitis, malaria, varicella, and sickle cell disease were excluded. Of the 151 children that were eligible for the study, 31 had been weaned, leaving 120 lactating mothers of children suffering from SAM included in our study (Fig 1A). 94.2% (113) of the samples were collected from mothers of marasmus cases while 5.8% (7) were collected from mothers of kwashiorkor cases. Of the 90 healthy children screened, 15 were excluded due to lack of parental consent, a parental withdrawal request or lack of sampling. 21 children presented clinical symptoms or diseases specifically pharyngitis, rhinitis, bronchitis, malaria, diarrhoea, or sickle cell disease and were therefore excluded. Of the 54 eligible children, 22 had been weaned, leading to a final cohort of 32 lactating mothers of healthy children retained for our study (Fig 1B).

**Table 1:** Baseline characteristics.

		SAM (n = 120)	CTL (n = 32)	*P-value*
**Locality**	Dakar	90	22	0.48^a^
	Fatick	30	10	
**Mother’s characteristics**				
**Age (years)**		27.3 ± 6.0	28.3 ± 6.6	0.64 ^b^
**Asymptomatic mother**		120 (100%)	32 (100%)	-
**Children’s characteristics**				
**Age (months)**		18 (12–24)	18 (9–24)	0.22 ^b^
**Sex (Female)**		76 (63.3%)	16 (50%)	0.17 ^a^
**Weight**		7.03 [6.2–8.5]	10.50 [9.4–16.0]	<10^−6^ ^c^
**Height**		76.25 ± 10.6	79.09 ± 9.8	0.071 ^b^
**WHZ**		-3.170 [-3.76; -2.56]	0.70 [0.34; 1.19]	<10^−5^ ^c^
**WAZ**		-3.225 [-3.98; -2.66]	0.28 [0.13; 0.67]	<10^−5^ ^c^
**HAZ**		-2.07 [-3.12; -1]	-0.49 [-1.16; 0.25]	<10^−5^ ^c^
**MUAC**		11.22 ± 0.55	13.69 ±0.85	0.00001 ^b^
**Fever**		98 (81.6%)	15 (46.9%)	10^−7^ ^a^
**Diarrhoea**		93 (77.5%)	0 (0%)	< 10^−7^ ^a^
**Oedema**		7 (5.8%)	0 (0%)	0.34 ^d^
**Death**		1 (0.8%)	0 (0%)	0.79^d^
**Diet**	Rice	120 (100%)	32 (100%)	-
	Millet	120 (100%)	32 (100%)	-
	Maize	30 (25.0%)	10 (31.2%)	0.48 ^a^
	Black eyed peas	42 (35.0%)	15 (46.9%)	0.22^a^
	Peanut	23 (19.2%)	32 (100%)	< 10^−8^ ^a^

^a^Chi square test

^b^bilateral Student test

^c^bilateral Mann-Whitney test

^d^bilateral Fisher exact test.

### Confirmed prevalence of *L*. *monocytogenes* in the breast milk of West African women

Using 16S amplicon sequencing, *L*. *monocytogenes* was detected in the breast milk of all the women included in this study. Real-time PCR was used to quantify *L*. *monocytogenes* after three different extraction protocols. Automated extraction made it possible to detect *L*. *monocytogenes* in 13/152 (8.5%) lactating women. To improve the detection of *L*. *monocytogenes*, two treatments were performed on the samples before automated extraction: proteinase K and deglycosylation. Proteinase K treatment increased the detection rate from 8.5% to 81.6% (124/152) of breast milk samples positive for *L*. *monocytogenes*. Deglycosylation and proteinase K treatment prior to automated extraction further improved the detection of *L*. *monocytogenes* to reach a proportion of 86.6% (132/152) of positive women. eglycosylation followed by proteinase K therefore markedly improved the detection of *L*. *monocytogenes* in breast milk using real-time PCR and was therefore the only protocol considered going forward.

Using v3v4 16S amplicon sequencing, all women included in this study were positive for *L*. *monocytogenes* (152/152 (100%) with a mean relative abundance of 0.29 (SD 0.34)). Interestingly, *Listeria grayi* was only detected in 10/132 (6.6%) of women. Therefore, the high prevalence of *Listeria* spp. detected in the breast milk of Senegalese lactating women consisted mostly of *L*. *monocytogenes*, a foodborne pathogen, at the species level. This high prevalence was reflected in the isolation of the 10 strains of *L*. *monocytogenes* from 10 samples, all collected from mothers of marasmus cases, the initial identification of which using MALDI-TOF MS was also confirmed through genome sequencing.

### Phylogenetic conservation of *Listeria monocytogenes* across anatomical niches and countries in West Africa

**Fig 2:**
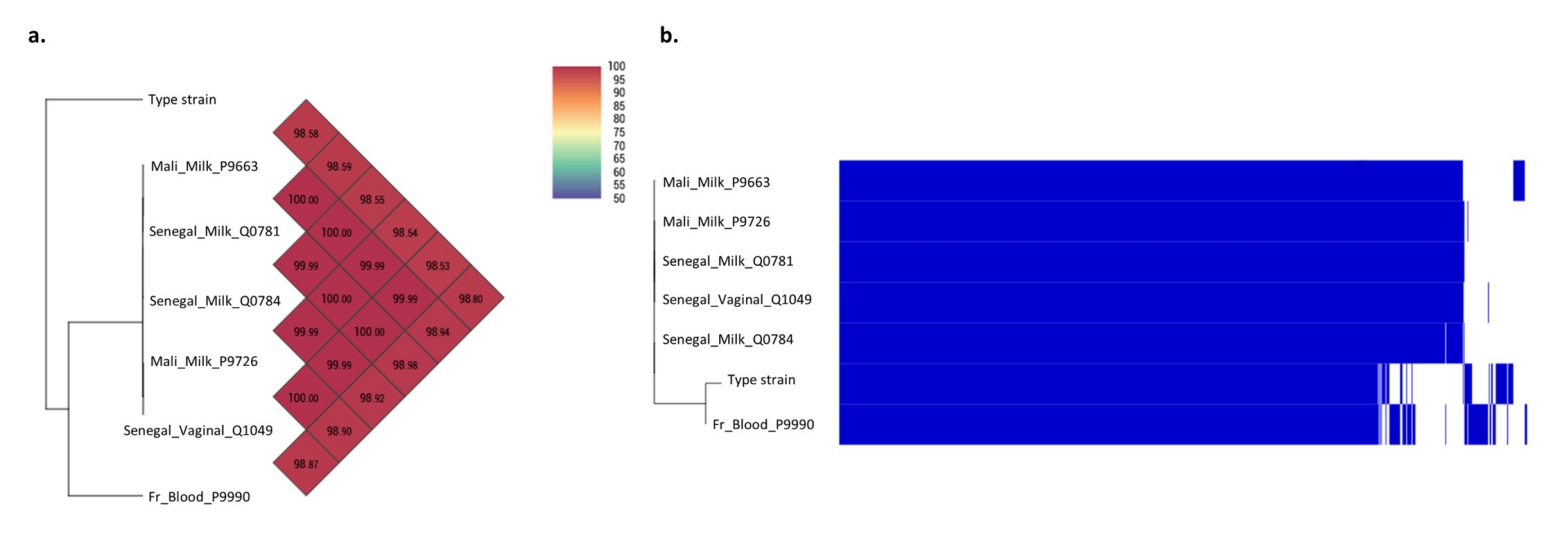
Endemic clonal circulation of *L*. *monocytogenes* in West Africa. (a) Genomic similarity estimated using Orthologous Average Nucleotide Identity Tool (OAT) software. (b) Pangenome carried out using the ROARY software.

As *L*. *monocytogenes* had an increased prevalence in several African datasets [18,19] and to clarify whether a hyperendemic clone was circulating in Africa, we analysed the pangenome of the eight strains previously isolated from human milk in Mali [18], the 10 strains isolated in this study, and the strain isolated from a vaginal sample from a woman in Senegal who had miscarried. The orthoANI analysis of the eight genomes of the strains isolated from the milk from Mali showed similarity percentages ranging from 99.99 and 100% between all strains, supporting the hypothesis of a single clone from Malian samples (Fig A in S1 Text). Similarly, the genomic comparison of the 10 strains isolated from Senegalese milk using orthoANI analysis showed similarity percentages between 99.99 and 100%, also supporting the hypothesis of a single Senegalese clone (Fig 2A). When comparing two breast milk strains from Mali (P9663 and P9726), two breast milk strains from Senegal (Q0781 and Q0784), the vaginal strain from Senegal (Q1049), and an isolated strain from a clinical sample (blood) in France in our laboratory (P9990) with the reference genome of the type strain of *L*. *monocytogenes*, orthoANI values ranged from 98.53 to 100% with all the African strains clearly clustered with orthoANI values ranging from 99.99 to 100%. Pangenome analysis using ROARY showed all African strains clustered and different from the type strain or the clinical strain (Fig 2B). Only one Malian strain (CSUR P9663) was slightly different, with an additional plasmid that was seemingly lost in the other strains (Fig 2B). All these analyses tend to show the existence of a single endemic clone of *L*. *monocytogenes* circulating in West Africa.

### Increased prevalence of *L*. *monocytogenes* in the milk of mothers of SAM children

**Fig 3:**
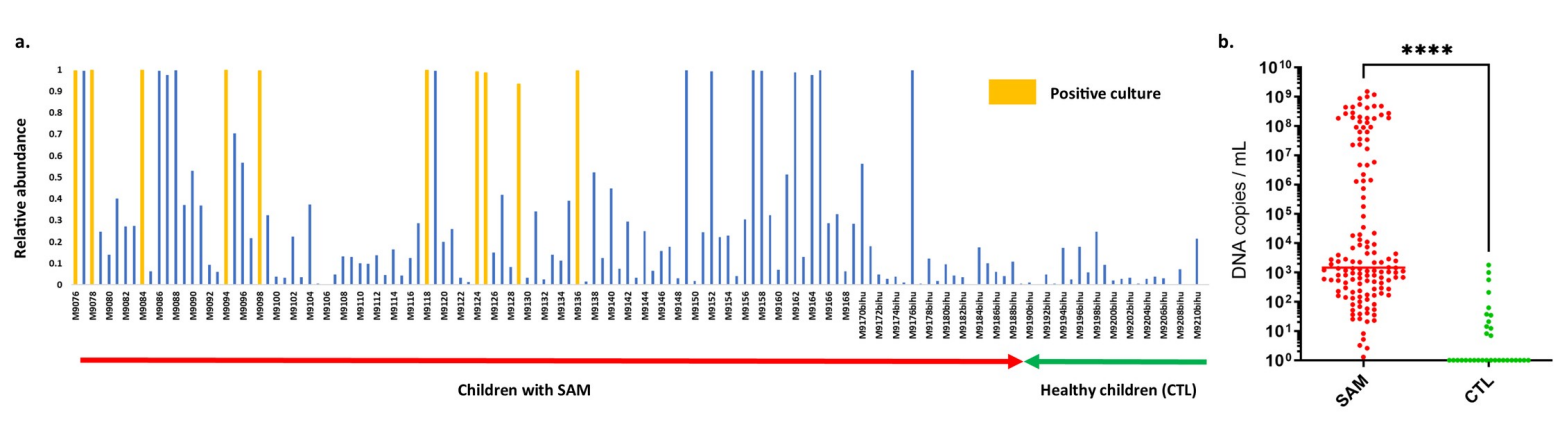
Detection of *Listeria monocytogenes* according to nutritional status. (a) Spike graph representing the relative abundance estimated according to the 16S amplicon sequencing. Samples that are culture positive are coloured yellow. (b) Comparison of the absolute concentration of *L*. *monocytogenes* assessed using qPCR between mothers of malnourished children (SAM) and those of healthy children (CTL).

Unlike when using 16S amplicon sequencing, the comparison based on the results of the real-time PCR analysis showed that all samples (120/120) from mothers of SAM children were positive for *L*. *monocytogenes*, while only 37.5% (12/32) of samples from mothers of healthy children were positive. These results highlight a significantly increased prevalence of *L*. *monocytogenes* in breast milk associated with SAM (100% (120/120) compared to 37.5% (12/32), bilateral mid-P-exact test, p = 10^−7^, S1 Table). 16S amplicon sequencing emphasised this observation with a significantly increased relative abundance of *L*. *monocytogenes* in the breast milk of mothers of SAM children (0.34 (SD 0.35) vs 0.05 (SD 0.07), p<0.0001, Fig 3A and 3B). Interestingly, no significant difference was observed between samples collected from marasmus cases and those collected from mothers of kwashiorkor cases (0.38 (SD 0.05) vs 0.48 (SD 0.18), p = 0.39). Conversely, the OTU matching *L*. *grayi* was less abundant in SAM compared to control samples (1.2e-5 (SD 5.8e-5) vs 9.5e-5 (SD 0.0003), p = 0.51, Fig B in S1 Text). In contrast to the 16S and PCR results, which show non-viable as well as viable organisms, the culture results were quite different. By culture all the *L*. *monocytogenes* strains were isolated from the breast milk of mothers of SAM children (10/120 vs 0/32 in mothers of healthy children, Barnard test, bilateral; p = 0.1 –unilateral; p = 0.036).

### Antimicrobial effect of the breast milk of mothers of healthy children against *L*. *monocytogenes*

**Fig 4:**
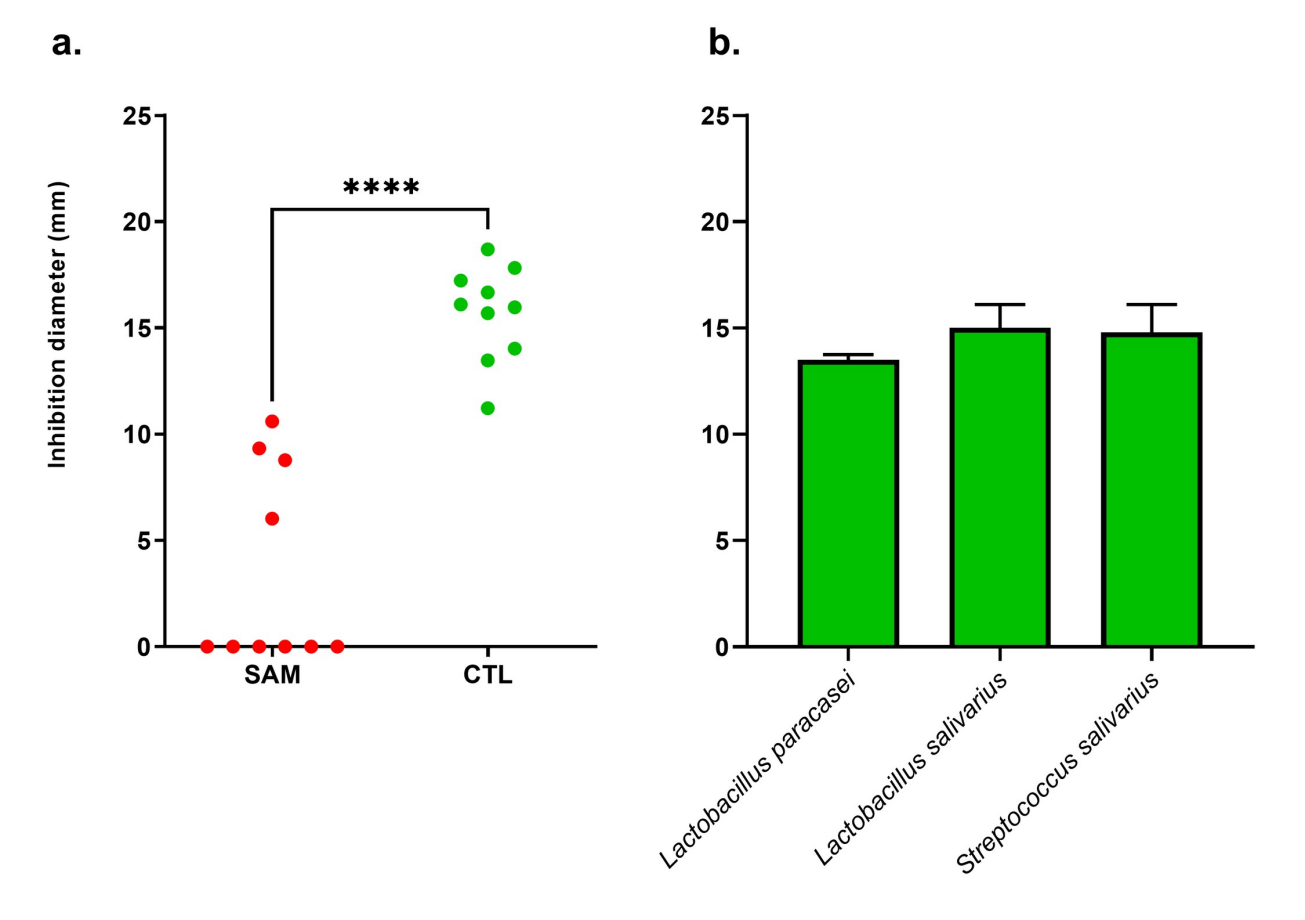
Antimicrobial activity of (a) breastmilk samples, ***: p < .0005, two-sided Mann-Whitney test, and (b) selected species isolated from breast milk samples against *L*. *monocytogenes* (strain CSUR Q0781 isolated in the present study from the breast milk of a mother with a severely malnourished child). n = 3 experiments, mean and standard deviation are reported. No anti-*L*. *monocytogenes* activity was found for the strains of *Bifidobacterium longum*, *Bifidobacterium breve*, *Lactobacillus brevis*, *Lactobacillus delbrueckii*, *Lactobacillus gasseri*, *Lactobacillus gastricus* or *Lactococcus lactis* isolated and tested in the present study.

Given the association between the increased prevalence and relative abundance of *L*. *monocytogenes* in the breast milk of lactating women and the nutritional status of their children, we assessed the antimicrobial activity of breast milk samples (10 randomly selected samples from each group) against *L*. *monocytogenes*. Growth inhibition was systematically obtained using breast milk from the lactating mothers of healthy children (10/10) while an inhibition was obtained with only 40% (4/10) of the breast milk from the lactating mothers of SAM children (bilateral Barnard test, p = 0.009). Moreover, the inhibition diameter of all the SAM children’s mothers was lower than the smallest inhibition diameter of the control mothers without any overlap (Fig 4A). Additionally, known probiotics previously used in the treatment of mastitis in the literature, namely *Lactobacillus paracasei*, *Lactobacillus salivarius* and *Streptococcus salivarius*, isolated here from mothers of healthy child, showed antimicrobial activity against *L*. *monocytogenes* with a species-specific effect (Fig 4B).

### Lactic acid bacteria enriched in the breast milk of mothers of healthy children

**Fig 5:**
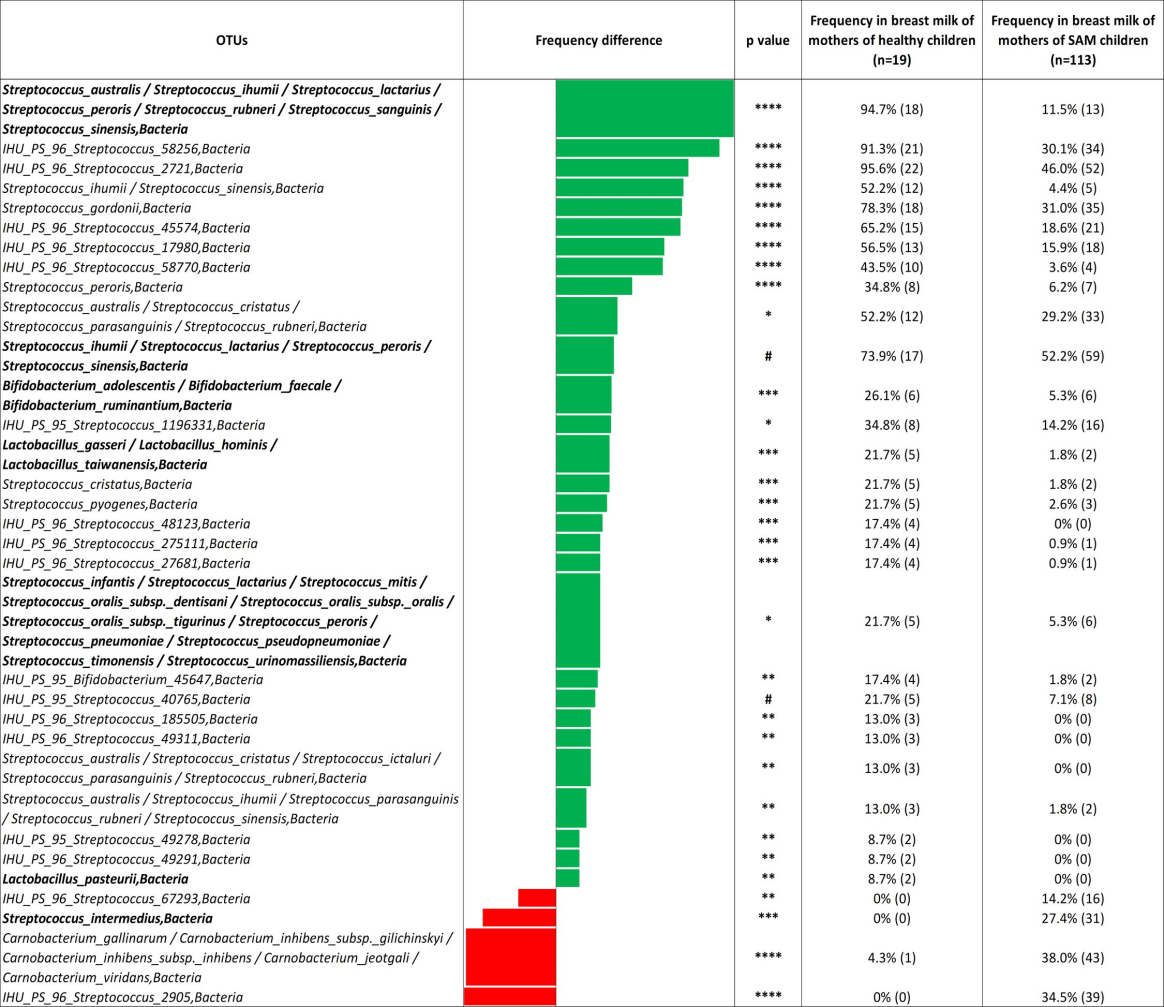
Comparison of the breast milk bacterial diversity assessed through 16S amplicon sequencing between the mothers of healthy children and those of severely malnourished children.

Because lactic acid bacteria (LAB) used as probiotics have also proved to have an antimicrobial activity against *L*. *monocytogenes*, the frequency of each OTU was estimated in each group (Fig 5). Most of the OTUs with a significant different frequency between SAM and healthy controls belonged to the *Streptococcus* genus (29/33 (87.9%)) suggesting that the role of *Streptococcus* in breast milk for the nutritional status of the children may have been overlooked. Several OTUs corresponding to the *Streptococcus* genus were enriched both in SAM and healthy controls (*Streptococcus* corresponded to 89.6% (26/29) OTUs enriched in the breast milk of mothers of healthy children versus 75% (3/4) in the breast milk of mothers of SAM children, p = 0.48).

Nevertheless, OTUs matching species associated with the health of children, for instance *Streptococcus lactarius* were enriched in healthy samples (94.7% (18/19) vs 11.5% (13/113), p<0.00001), as for *Streptococcus infantis* (21.7% (5/19) vs 5.3% (6/113), p < 0.05). Conversely, *Streptococcus intermedius*, associated with brain abscesses in children [27], was only detected in the breast milk consumed by SAM children (27.4% (31/113) vs 0% in healthy children, p = 0.004). Two OTUs matching the *Bifidobacterium* genus, including *Bifidobacterium adolescentis* (26.1% (6/19) vs 5.3% (6/113), p < 0.0005) and an unidentified species (IHU_PS_*Bifidobacterium*_45647, 17.4% (4/19) vs 1.8% (2/113), p < 0.005) and two OTUs matching the *Lactobacillus* genus, *L*. *gasseri* (21.7% (5/19) vs 1.8% (2/113), p < 0.0005) and *L*. *pasteurii* (8.7% (2/19) vs 0%, p < 0.005) were enriched in the breast milk consumed by healthy children (Fig 5).

## Discussion

This work provides confirmatory evidence of the very high prevalence of *L*. *monocytogenes* in the breast milk of west African women that we first reported in Mali [18]. Surprisingly, this incidence has been ignored and had never been evaluated in lactating mothers, despite the fact that *L*. *monocytogenes* is known to circulate in the milk of mammals typically consumed by humans [28,29], and is even one of the major sources of human foodborne infections recognised to date [19]. However, this high prevalence was only revealed subsequently to a proteic lysis and deglycosylation of the milk samples, probably due to the high content in complex proteins and human milk oligosaccharides [30]. This explains the highly variable prevalence of *L*. *monocytogenes* detected depending on the DNA extraction protocol and highlights the importance of an appropriate DNA extraction.

These results should spark a great deal of work around the world, particularly in Africa, to evaluate the prevalence of *L*. *monocytogenes* in larger cohorts and determine the role of *L*. *monocytogenes* in neonatal pathology. Furthermore, in this study, we highlight a significantly increased prevalence of *L*. *monocytogenes* in the milk of lactating mothers of SAM children compared to that of lactating mothers of healthy children, suggesting an implication of *L*. *monocytogenes* in human breast milk in the pathogenesis of SAM. Indeed, the gut microbiota of SAM children presents the particularity of a low prevalence of lactic acid bacteria [7,31] which are inhibitors of *L*. *monocytogenes*, particularly *Lactobacillus* species [32,33]. In this study, we have confirmed the activity of lactic acid bacteria against *L*. *monocytogenes*, which may prevent the risk associated with this foodborne pathogen in well-fed children with a non-dysbiotic microbiota. This antagonism between lactic acid bacteria and *L*. *monocytogenes* is of the utmost importance since it has been shown that oral administration of *Lactobacillus* species was effective against mastitis in lactating women [34–36] as well as in cows for the prevention of mastitis and brain abscesses [37]. There is a verified passage of orally administered *Lactobacillus salivarius* in the milk of lactating mothers and cows [36] the efficacy of which to inhibit *L*. *monocytogenes* was shown here. Fermented dairy products or probiotics containing *L*. *paracasei*, are already widely marketed and consumed in countries with a low prevalence of malnutrition such as Western countries and Japan (*L*. *paracasei* strain Shirota). *L*. *salivarius*, *L*. *paracasei and S*. *salivarius* or other antagonistic strains could eliminate *L*. *monocytogenes* from the milk of lactating women and preserve the expected prevalence in lactic acid bacteria that may be missing in SAM children [7,10,31].

The increased relative abundance of *L*. *monocytogenes* in the milk of the mothers of SAM children hints towards a dysbiosis of their breast milk microbiota. Future studies should therefore further investigate the composition of the breast milk and gut microbiota of mothers of SAM children and characterize the associated dysbiosis. The presence of *L*. *monoctytogenes* in the breast milk of African lactating mothers underlines a source of infection in children that has been completely neglected to date. Nonetheless, the efficacy of carefully selected strains of lactic acid bacteria, already known for their safety, against *L*. *monocytogenes* provides the possibility of inexpensive and simple therapeutic interventions against *L*. *monocytogenes* infection and hopefully the correction of undernutrition-related microbiota disruption.

## Supporting information

S1 STROBE Checklist (DOCX)

S1 Table Frequency of *Listeria monocytogenes* detection in lactating mothers in Senegal depending on the method. (DOCX)

S1 Text **Fig A.** Pangenome of the strains of *Listeria monocytogenes* isolated from Malian breast milk samples carried out using the ROARY software. **Fig B.** Spike graphs representing the relative abundance of *Listeria grayi* estimated according to the 16S amplicon sequencing.
(DOCX)

S1 Data Participants characteristics and results of culture, qPCR and 16S amplicon sequencing. (XLSX)

## References

[1] Pravana NK, Piryani S, Chaurasiya SP, Kawan R, Thapa RK, Shrestha S. Determinants of severe acute malnutrition among children under 5 years of age in Nepal: a community-based case-control study. BMJ Open. 2017;7: e017084. doi: 10.1136/bmjopen-2017-017084 28851796 PMC5724075

[2] Research Institute (IFPRI) IFP. Global Nutrition Report 2016 From Promise to Impact Ending Malnutrition by 2030. 0 ed. Washington, DC: International Food Policy Research Institute; 2016. doi: 10.2499/9780896295841

[3] Schwinger C, Golden MH, Grellety E, Roberfroid D, Guesdon B. Severe acute malnutrition and mortality in children in the community: Comparison of indicators in a multi-country pooled analysis. PLOS ONE. 2019;14: e0219745. doi: 10.1371/journal.pone.0219745 31386678 PMC6684062

[4] Bichard A, Leturque H. Intégration de la nutrition dans les politiques et programmes des secteurs contributeurs (2015); rapport de synthèse d’une étude régionale portant sur l’Afrique de l’ouest—Recherche Google. [cited 18 Nov 2020]. Available from: https://www.actioncontrelafaim.org/wp-content/uploads/2018/01/acf_iramintegrationnutrition.pdf.

[5] Hammond W, Badawi AE, Deconinck H. Detecting severe acute malnutrition in children under five at scale. The Challenges of Anthropometry to Reach the Missed Millions. Ann Nutr Disord Ther. 2016;3:1–5.

[6] Ahmed T, Rahman S, Cravioto A. Oedematous malnutrition. Indian J Med Res. 2009;130: 651+654. 20090122

[7] Million M, Diallo A, Raoult D. Gut microbiota and malnutrition. Microb Pathog. 2016. doi: 10.1016/j.micpath.2016.02.003 26853753

[8] Smith MI, Yatsunenko T, Manary MJ, Trehan I, Mkakosya R, Cheng J, et al. Gut Microbiomes of Malawian Twin Pairs Discordant for Kwashiorkor. Science. 2013;339:548–554. doi: 10.1126/science.1229000 23363771 PMC3667500

[9] Million M, Tidjani Alou M, Khelaifia S, Bachar D, Lagier J-C, Dione N, et al. Increased Gut Redox and Depletion of Anaerobic and Methanogenic Prokaryotes in Severe Acute Malnutrition. Sci Rep. 2016;6: 26051. doi: 10.1038/srep26051 27183876 PMC4869025

[10] Tidjani Alou M, Million M, Traore SI, Mouelhi D, Khelaifia S, Bachar D, et al. Gut Bacteria Missing in Severe Acute Malnutrition, Can We Identify Potential Probiotics by Culturomics? Front Microbiol. 2017;8. doi: 10.3389/fmicb.2017.00899 28588566 PMC5440526

[11] Pham T-P-T, Alou MT, Golden MH, Million M, Raoult D. Difference between kwashiorkor and marasmus: Comparative meta-analysis of pathogenic characteristics and implications for treatment. Microb Pathog. 2021;150:104702. doi: 10.1016/j.micpath.2020.104702 33359074

[12] Pham T-P-T, Tidjani Alou M, Bachar D, Levasseur A, Brah S, Alhousseini D, et al. Gut Microbiota Alteration is Characterized by a Proteobacteria and Fusobacteria Bloom in Kwashiorkor and a Bacteroidetes Paucity in Marasmus. Sci Rep. 2019;9:9084. doi: 10.1038/s41598-019-45611-3 31235833 PMC6591176

[13] Williams CD. A nutritional disease of childhood associated with a maize diet. Arch Dis Child. 1933;8:423–433. doi: 10.1136/adc.8.48.423 21031941 PMC1975318

[14] Ballard O, Morrow AL. Human Milk Composition: Nutrients and Bioactive Factors. Pediatr Clin North Am. 2013;60:49–74. doi: 10.1016/j.pcl.2012.10.002 23178060 PMC3586783

[15] Togo A, Dufour J-C, Lagier J-C, Dubourg G, Raoult D, Million M. Repertoire of human breast and milk microbiota: a systematic review. Future Microbiol. 2019;14:623–641. doi: 10.2217/fmb-2018-0317 31025880

[16] Murphy K, Curley D, O’Callaghan TF, O’Shea C-A, Dempsey EM, O’Toole PW, et al. The Composition of Human Milk and Infant Faecal Microbiota Over the First Three Months of Life: A Pilot Study. Sci Rep. 2017;7:40597. doi: 10.1038/srep40597 28094284 PMC5240090

[17] Svabić-Vlahović M, Pantić D, Pavićić M, Bryner JH. Transmission of Listeria monocytogenes from mother’s milk to her baby and to puppies. Lancet Lond Engl. 1988;2:1201. doi: 10.1016/s0140-6736(88)90276-0 2903417

[18] Togo AH, Dubourg G, Camara A, Konate S, Delerce J, Andrieu C, et al. Listeria monocytogenes in human milk in Mali: A potential health emergency. J Infect. 2020;80:121–142. doi: 10.1016/j.jinf.2019.09.008 31560881

[19] Charlier C, Perrodeau É, Leclercq A, Cazenave B, Pilmis B, Henry B, et al. Clinical features and prognostic factors of listeriosis: the MONALISA national prospective cohort study. Lancet Infect Dis. 2017;17:510–519. doi: 10.1016/S1473-3099(16)30521-7 28139432

[20] von Elm E, Altman DG, Egger M, Pocock SJ, Gøtzsche PC, Vandenbroucke JP. Strengthening the reporting of observational studies in epidemiology (STROBE) statement: guidelines for reporting observational studies. BMJ. 2007;335:806–808. doi: 10.1136/bmj.39335.541782.AD 17947786 PMC2034723

[21] World Health Organization, UNICEF. WHO child growth standards and the identification of severe acute malnutrition in infants and children: a joint statement by the World Health Organization and the United Nations Children’s Fund. 2009. Available from: http://www.ncbi.nlm.nih.gov/books/NBK200775/. 24809116

[22] WHO Anthro Survey Analyser and other tools. [cited 19 Dec 2020]. Available from: https://www.who.int/tools/child-growth-standards/software.

[23] Dridi B, Henry M, El Khéchine A, Raoult D, Drancourt M. High prevalence of Methanobrevibacter smithii and Methanosphaera stadtmanae detected in the human gut using an improved DNA detection protocol. PloS One. 2009;4:e7063. doi: 10.1371/journal.pone.0007063 19759898 PMC2738942

[24] Angelakis E, Bachar D, Henrissat B, Armougom F, Audoly G, Lagier J-C, et al. Glycans affect DNA extraction and induce substantial differences in gut metagenomic studies. Sci Rep. 2016;6:26276. doi: 10.1038/srep26276 27188959 PMC4870698

[25] Bellali S, Lagier J-C, Million M, Anani H, Haddad G, Francis R, et al. Running after ghosts: are dead bacteria the dark matter of the human gut microbiota? Gut Microbes. 2021;13:1–12. doi: 10.1080/19490976.2021.1897208 33757378 PMC7993147

[26] Barnard GA. A New Test for 2 × 2 Tables. Nature. 1945;156:177. doi: 10.1038/156177a0

[27] Yakut N, Kadayifci EK, Karaaslan A, Atici S, Akkoc G, Ocal Demir S, et al. Braın abscess due to Streptococcus intermedius secondary to mastoiditis in a child. SpringerPlus. 2015;4:809. doi: 10.1186/s40064-015-1608-0 26722629 PMC4689728

[28] Quigley L, O’Sullivan O, Stanton C, Beresford TP, Ross RP, Fitzgerald GF, et al. The complex microbiota of raw milk. FEMS Microbiol Rev. 2013;37:664–698. doi: 10.1111/1574-6976.12030 23808865

[29] Castro H, Ruusunen M, Lindström M. Occurrence and growth of Listeria monocytogenes in packaged raw milk. Int J Food Microbiol. 2017;261:1–10. doi: 10.1016/j.ijfoodmicro.2017.08.017 28850852

[30] Andreas NJ, Kampmann B, Mehring Le-Doare K. Human breast milk: A review on its composition and bioactivity. Early Hum Dev. 2015;91:629–635. doi: 10.1016/j.earlhumdev.2015.08.013 26375355

[31] Subramanian S, Huq S, Yatsunenko T, Haque R, Mahfuz M, Alam MA, et al. Persistent gut microbiota immaturity in malnourished Bangladeshi children. Nature. 2014;510:417–421. doi: 10.1038/nature13421 24896187 PMC4189846

[32] Million M, Angelakis E, Drissi F, Raoult D. Occam’s razor and probiotics activity on Listeria monocytogenes. Proc Natl Acad Sci U S A. 2013;110:E1. doi: 10.1073/pnas.1218418110 23271812 PMC3538255

[33] Archambaud C, Nahori M-A, Soubigou G, Bécavin C, Laval L, Lechat P, et al. Impact of lactobacilli on orally acquired listeriosis. Proc Natl Acad Sci U S A. 2012;109:16684–16689. doi: 10.1073/pnas.1212809109 23012479 PMC3478606

[34] Jiménez E, Fernández L, Maldonado A, Martín R, Olivares M, Xaus J, et al. Oral Administration of Lactobacillus Strains Isolated from Breast Milk as an Alternative for the Treatment of Infectious Mastitis during Lactation. Appl Environ Microbiol. 2008;74:4650–4655. doi: 10.1128/AEM.02599-07 18539795 PMC2519365

[35] Arroyo R, Martín V, Maldonado A, Jiménez E, Fernández L, Rodríguez JM. Treatment of infectious mastitis during lactation: antibiotics versus oral administration of Lactobacilli isolated from breast milk. Clin Infect Dis Off Publ Infect Dis Soc Am. 2010;50:1551–1558. doi: 10.1086/652763 20455694

[36] Fernández L, Cárdenas N, Arroyo R, Manzano S, Jiménez E, Martín V, et al. Prevention of Infectious Mastitis by Oral Administration of Lactobacillus salivarius PS2 During Late Pregnancy. Clin Infect Dis Off Publ Infect Dis Soc Am. 2016;62:568–573. doi: 10.1093/cid/civ974 26611780

[37] Soleimani NA, Kermanshahi RK, Yakhchali B, Sattari TN. Antagonistic activity of probiotic lactobacilli against Staphylococcus aureus isolated from bovine mastitis. Afr J Microbiol Res. 2010;4:2169–2173. doi: 10.5897/AJMR.9000040

