# Supplementary material for: A Listeria monocytogenes clone in human breast milk associated with severe acute malnutrition in West Africa: A multicentric case-controlled study
Source: PLoS Negl Trop Dis. 2021 Jun 29;15(6):e0009555. doi: 10.1371/journal.pntd.0009555 (PMC8291692; doi:10.1371/journal.pntd.0009555)
Supplement: S1 Table — (DOCX) [file pntd.0009555.s002.docx]

**S1 Table. Frequency of *Listeria monocytogenes* detection in lactating mothers in Senegal according to methods of detection**

|  | *All samples (n=152)* | *SAM (n=120)* | *CTL (n=32)* | *p-value (SAM vs CTL)* |
| --- | --- | --- | --- | --- |
| *Culture* | *10 (6.6%)* | *10 (8.3%)* | *0 (0%)* | *0.10* |
| *Quantitative PCR (all extraction methods^a^)* | *132 (86.8%)* | *120 (100%)* | *12 (37.5%)* | *10^-7^* |
| *Automated extraction^a^* | *13 (8.5%)* | *13 (10.8%)* | *0 (0%)* | *0.047* |
| *Proteinase K treatment prior to automated extraction^a^* | *124 (81.6%)* | *120 (100%)* | *4 (12.5%)* | *10^-7^* |
| *Proteinase K and deglycosylation treatment prior to automated extraction^a^* | *132 (86.8%)* | *120 (100%)* | *12 (37.5%)* | *10^-7^* |
|  |  |  |  |  |
| *v3v4 16S amplicon sequencing* | *152 (100%)* | *120 (100%)* | *32 (100%)* | *-* |

SAM: severe acute malnutrition, CTL: healthy controls, ^a^See methods for detailed DNA extraction protocols used prior to quantitative PCR.
