# Supplementary material for: A Listeria monocytogenes clone in human breast milk associated with severe acute malnutrition in West Africa: A multicentric case-controlled study
Source: PLoS Negl Trop Dis. 2021 Jun 29;15(6):e0009555. doi: 10.1371/journal.pntd.0009555 (PMC8291692; doi:10.1371/journal.pntd.0009555)

**Fig A: Pangenome of the strains of *Listeria monocytogenes* isolated from Malian breast milk samples carried out using the ROARY software**


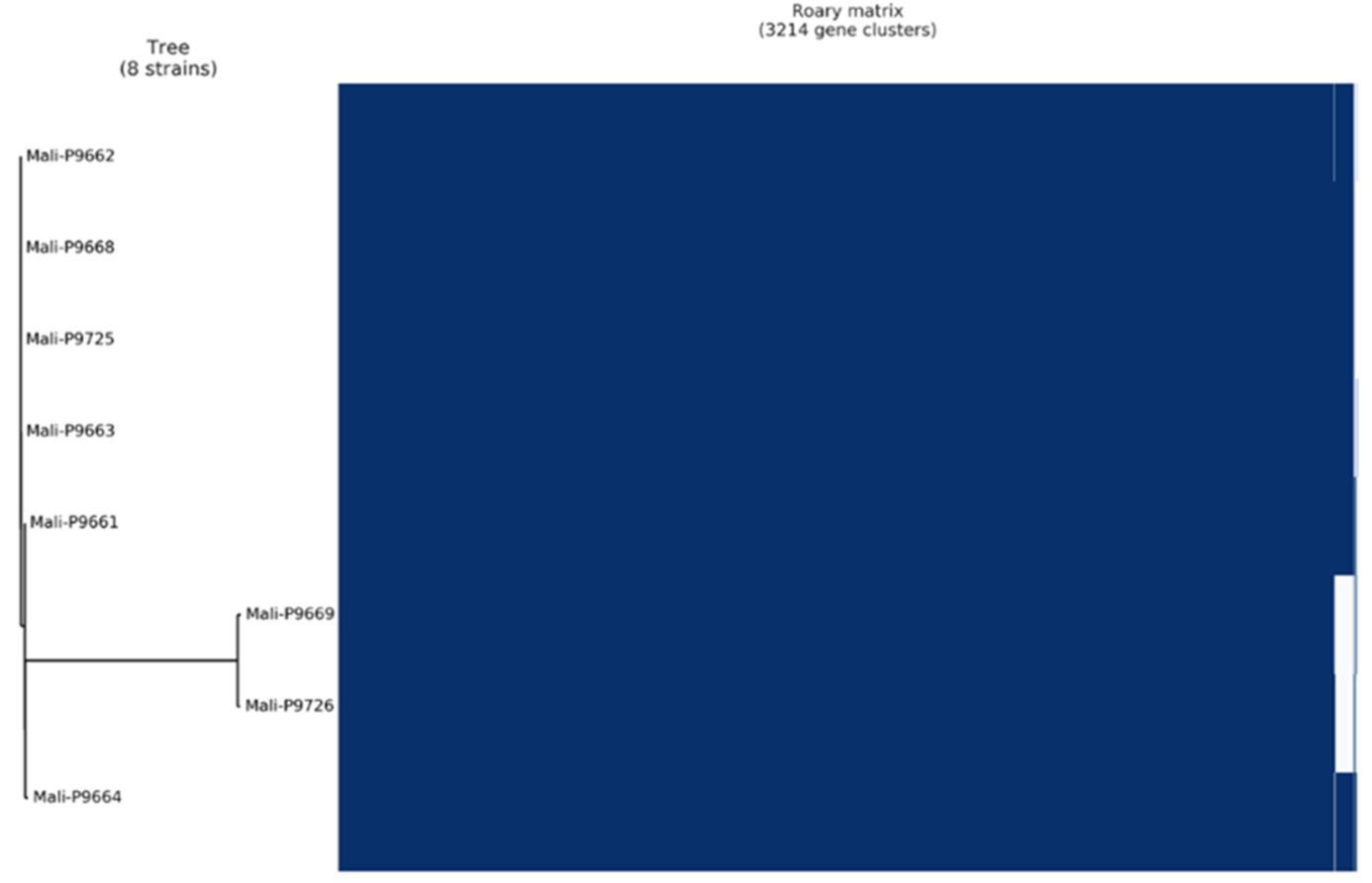


**Fig B. Spike graphs representing the relative abundance of *Listeria grayi* estimated according to the 16S amplicon sequencing**


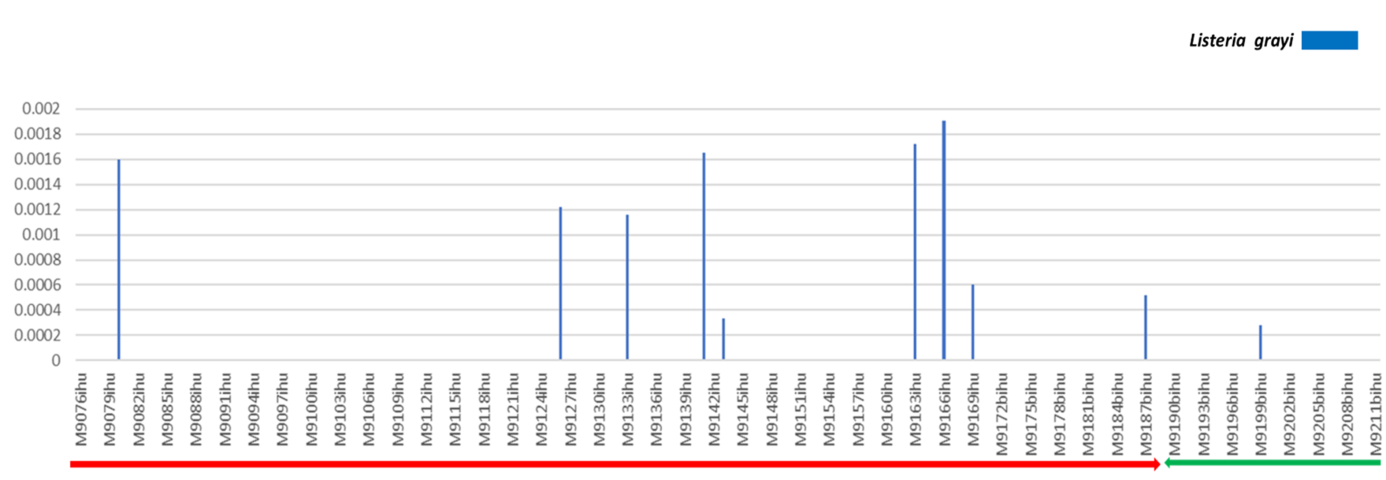

Supplement: S1 Text — Fig A. Pangenome of the strains of Listeria monocytogenes isolated from Malian breast milk samples carried out using the ROARY software. Fig B. Spike graphs representing the relative abundance of Listeria grayi estimated according to the 16S amplicon sequencing. (DOCX) [file pntd.0009555.s003.docx]
